# Microbiota’s Role in Diet-Driven Alterations in Food Intake: Satiety, Energy Balance, and Reward

**DOI:** 10.3390/nu13093067

**Published:** 2021-08-31

**Authors:** Allison W. Rautmann, Claire B. de La Serre

**Affiliations:** Department of Nutritional Sciences, University of Georgia, Athens, GA 30605, USA; awr76051@uga.edu

**Keywords:** microbiome, food intake, vagus, CCK, hypothalamus, reward

## Abstract

The gut microbiota plays a key role in modulating host physiology and behavior, particularly feeding behavior and energy homeostasis. There is accumulating evidence demonstrating a role for gut microbiota in the etiology of obesity. In human and rodent studies, obesity and high-energy feeding are most consistently found to be associated with decreased bacterial diversity, changes in main phyla relative abundances and increased presence of pro-inflammatory products. Diet-associated alterations in microbiome composition are linked with weight gain, adiposity, and changes in ingestive behavior. There are multiple pathways through which the microbiome influences food intake. This review discusses these pathways, including peripheral mechanisms such as the regulation of gut satiety peptide release and alterations in leptin and cholecystokinin signaling along the vagus nerve, as well as central mechanisms, such as the modulation of hypothalamic neuroinflammation and alterations in reward signaling. Most research currently focuses on determining the role of the microbiome in the development of obesity and using microbiome manipulation to prevent diet-induced increase in food intake. More studies are necessary to determine whether microbiome manipulation after prolonged energy-dense diet exposure and obesity can reduce intake and promote meaningful weight loss.

## 1. Introduction

The gut microbiota is a collection of over 10^13^ microorganisms, including bacteria and fungi, that inhabit the gastrointestinal (GI) tract and plays a key role in regulating host physiology, particularly GI function and energy homeostasis [1,2]. The microbiome is a relatively stable community of microbes in the individual [3]. In response to the burgeoning obesity epidemic, research has focused on personal and environmental factors that might influence weight status. The discovery that in both humans and rodent models, obese individuals have a distinct microbiome profile compared to their lean counterparts, with an increased capacity to harvest energy from ingested food, has fueled over 15 years of research [2]. Microbiota is vital for proper GI function, as it is implicated in vitamin synthesis, digestion and metabolism of carbohydrate and other dietary components [4], and development and function of the GI immune system [5]. Gut microbes have also been shown to influence the function of other peripheral organs, as well as the central nervous system (CNS), throughout development and the lifespan [6,7]. The importance of the gut microbiota in regulating host biology is evident from gnotobiotic studies: animals born germ-free (GF) present with altered intestinal, metabolic, and neural physiology [8,9].

Recently, advances in sequencing technologies have allowed us to more comprehensively and thoroughly assess microbiota composition and its relation with disease states. Adverse changes in composition have been associated with an array of pathologies, including autoimmune diseases, neurological conditions, and metabolic disorders such as obesity and diabetes [10,11,12,13]. It is, however, important to note that any environmental modification is likely to impact microbiome composition, and differences in bacterial makeup associated with pathologies do not equate to a causal link between microbial changes and pathological development.

There is accumulating evidence supporting a role for an individual’s microbiota in regulation of food intake through both peripheral and central mechanisms. Peripherally, bacteria and their metabolites interact with vagal afferent neurons (VANs), which transmit information about intestinal contents to the nucleus of the solitary tract (NTS) [14]. The microbiome influences gut–brain satiety signaling via modulation of gut peptide release [15] as well as sensitivity to satiety peptides (such as cholecystokinin, or CCK) and the energy storage hormone leptin [14]. Changes in microbiome composition have also been reported to affect the structural integrity of the gut–brain axis [16]. Centrally, unfavorable microbiome composition is associated with inflammation of key regions involved in the regulation of feeding, particularly the NTS and the hypothalamus [17,18]. Further, there is emerging evidence that certain taxa of bacteria play a role in modulating reward circuitry and motivation [19,20]. The purpose of this review is to describe the microbiota’s influence on food intake through the aforementioned mechanisms, including recent developments in the relationship between microbiome, reward, and eating behavior.

## 2. Energy-Dense Diets Alters Gut–Brain Communication and Regulation of Feeding

Chronic intake of energy-dense food has been linked to excessive weight gain [21]. Despite homeostatic signals that act protectively against food overconsumption, chronic intake of palatable, high-energy diets alters the physiological response to food and favors overeating. Ultimately, this results in increased body weight (BW) and fat deposition. Specifically, sensitivity to hedonic cues is altered, while homeostatic signals of meal termination are dampened [22].

The vagus nerve is a direct pathway that carries post-ingestive feedback from the gut to the brain [23]. Mechano- and chemosensitive VANs respond to the nutrient composition of ingested food to regulate meal size [24]. VANs terminate in the NTS, where postprandial signals increase neuronal activity [25]. In addition, VANs project to limbic brain regions, and this gut–reward circuit is sufficient and necessary for meal termination [23]. Chronic consumption of a high-fat (HF) diet reduces VAN sensitivity to tension [26,27], satiation hormones (e.g., CCK) [26,28,29,30,31,32,33], and intestinal nutrients [34,35,36,37]. As such, diet-induced disruption of vagal signaling coincides with the onset of hyperphagia [38]. In addition, diet-induced obese (DIO) rats also exhibit significantly decreased postprandial neuronal activation in the NTS compared to lean animals [29,37].

Other neuronal networks involved in the regulation of feeding are also altered by chronic HF consumption. Leptin is a key adiposity signal, with amounts produced proportional to the amount of fat stored in the body [39]. Hypothalamic leptin signaling is disrupted during chronic HF feeding [40], with increased expression of suppressor of cytokine signaling 3 (SOCS3) and decreased phosphorylated signal transducer and activator of transcription 3 (STAT3) in the arcuate nucleus [41]. Pro-opiomelanocortin (POMC) neurons in the hypothalamus in a normal physiological state are activated by leptin [42] to ultimately decrease food intake via the production of α-melanocyte-stimulating hormone (α-MSH) [43,44]. Thus, HF-induced disruption of leptin can directly alter hypothalamic inhibition of food intake. Another neuronal system altered by HF intake is the dopaminergic reward system. Food’s hedonic value is an important factor in food consumption, and increased motivation for food intake is linked to obesity [45]. Palatable foods initially have a higher reward value [46], while as obesity progresses, reductions in reward signaling emerge and lead to compensatory overeating [47]. Among regions involved in the mesolimbic dopaminergic system, the nucleus accumbens (NAc) and striatum exhibit decreased dopamine release in rodents with long-term exposure to a HF diet [48].

### Microbiome Alterations Seen with Energy-Dense Feeding

Dietary intake is a major and easily modifiable determinant of an individual’s microbiota composition; other factors include age and genetics [49]. In humans, both short- and long-term intake of specific macronutrients, as well as fibers and other plant foods, are correlated with abundance distribution of specific bacterial taxa present in the GI tract [49]. Obesity is associated with changes in microbiome composition. While the vast majority of the composition is specific to the individual, small-scale studies have found that obesity has been associated with an increased ratio of Firmicutes to Bacteroidetes, the two main phyla present in the GI tract [12]. Conversely, weight loss through caloric restriction has led to an increase in Bacteroidetes abundance, whether that restriction was through a carbohydrate- or fat-restricted diet plan [12]. Similar results are observed in rodent models of DIO, with the addition of a bloom in the pro-inflammatory Proteobacteria sometimes reported in humans with obesity or type 2 diabetes [50]. In rats, 8 weeks of 45% HF-feeding is associated with decreased bacterial α-diversity, a measure of the variety of bacterial taxa colonizing the gut, and increased relative abundances in the Firmicutes orders *Clostridiales* [51], in particular, the *Dorea* genus, and *Erysipelotrichiales* (*Erysipelotrichaceae* family) [52]. Three weeks of 60% HF-feeding has a similar effect on microbiome composition in rats, with increases in relative abundances of several Firmicutes families, including *Streptococcaceae*, *Erysipelotrichaceae*, *Lachnospiraceae* (*Dorea* genus), *Peptococcaceae*, and *Staphylococcaceae*, as well as Proteobacteria families *Desulfovibrionaceae* and *Enterobacteriaceae* [16]. In rats, a mere 7 days of 60% HF-feeding is associated with decreased abundance of the Bacteroidetes orders *Bacteroidales* (*Prevotella* genus) and *Sphingobacteriales*, and increased Firmicutes order *Erysipelotrichales*, and several Proteobacteria orders, including *Rhodocyclales* and *Altermondales*, among others [53]. Diets high in sugars also affect gut microbiome composition, with alterations in the Firmicutes to Bacteroidetes ratio sometimes being reported [17,54], but not always [55]. Different results have also been observed with respect to α-diversity [17,54,55,56]. In a study comparing high-glucose, high-fructose, and HF diets in mice, researchers found that all three similarly decreased diversity, decreased Bacteroidetes abundance (specifically *Muribaculum* spp.) and increased Proteobacteria abundance (specifically *Desulfovibrio* spp.); however, diets high in sugars led to a significant increase in *Akkermansia muciniphila* abundance compared to a HF diet [54]. When compared to an unrefined chow diet, both refined low-fat, high-sugar (LFHS) and HF, high-sugar (HFHS) diet consumption in rats result in decreased diversity and significant alterations in relative abundances within 1 week of feeding [17]. The LFHS diet increases Firmicutes, particularly *Ruminococcaceae* and *Lachnospiraceae*, as well as Proteobacteria genera *Sutterella* and *Bilophila*, and decreases Bacteroidetes abundances, though changes are more pronounced with HFHS-feeding. [17]. A mere daily 2 h access to HFHS pellets alters gut microbiome composition in chow-fed rats with increased relative abundances of *Lachnospiraceae*, *Ruminoccoceae*, and *Erysipelotrichaceae* families [56].

In humans, specific dietary patterns and components have been reported to affect bacterial taxa relative abundances. Obese humans switched to a strict vegan diet low in fat and high in fiber display an increase in abundance of Bacteroidetes over a 4-week period [57]. Self-classified vegans tend to have lower *Bifidobacterium* spp., *Escherichia coli*, and *Enterobacteriaceae* spp. when compared to vegetarian and omnivorous humans [58]. Two recent studies published in 2021 associated specific dietary components with microbial taxa. In Japanese monozygotic twins, significant associations are found between *Lachnospiraceae* species: *Lachnospira* and *Lachnospiraceae* UCG-008 negatively correlate with protein intake and saturated fat intake, respectively, while the *Lachnospiraceae* ND3007 group correlates positively with total fat intake [59]. A population study of 1920 Chinese adults found that a calculated healthy diet score, based on intakes of fruit, vegetables, seafood, nuts/legumes, refined grains, red meat, and processed meat, is associated with increased abundances within Firmicutes and Actinobacteria, particularly the genera *Coprococcus* and *Bifidobacterium*. Dairy is positively associated with the family *Bifidocacteraceae* and genus *Bifidobacterium*; seafood with families *Alcaligenaceae* and *Desulfovibrionaceae*; and nuts and legumes with the phyla Proteobacteria. Inverse associations are found with processed meat and the family *Lachnospiraceae*, while it was positively associated with *Fusobacteriaceae* and *Acinetobacter* under Proteobacteria [60].

There is evidence that Western-type diet-driven changes in microbiome composition negatively affect host metabolism and energy homeostasis. Studies using microbiota-depleted and GF rodent models have established a relationship between diet-driven dysbiosis and excessive weight gain. GF mice have been shown to exhibit resistance to weight gain when fed a HFHS diet that leads to increased adiposity in a conventional mouse, showing that microbiota is necessary for DIO [61]. Conversely, GF rats and mice colonized with fecal and cecal contents from conventional HFHS-fed animals display a significant increase in BW compared to rodents colonized with chow-fed animal microbiome [16,62]. Similar results have been observed when GF animals are re-colonized with GI contents from a genetically obese donor [2] or from an obese human donor [63]. We have successfully replicated these findings in an antibiotic depletion model [16]. These studies establish that an “obese microbiome” from a host that is obese, or “HF-type microbiome” from a host fed a HF diet, is sufficient to alter energy homeostasis and affect BW regulation, at least in the short term. The GF studies cited here do not extend past 5 weeks post-colonization [2,16,63]. While there is evidence that an individual’s microbiota composition may affect energy harvest [2], storage [18], and utilization [64], a major effect on BW may be driven by changes in regulation of energy intake.

Microbiota-depleted rats colonized with a HF-type microbiome have been shown to significantly increase weekly food intake compared to rats colonized with a chow-type microbiome [16]. Conversely, modulation of the microbiome via supplementation of anti-, pro-, or prebiotics impacts weight and intake. In rats fed a 60% HF diet, administration of minocycline, a broad-spectrum antibiotic, lessens microbiome alterations and significantly reduces food intake [53]. In this experiment, 3 weeks of antibiotic administration normalizes HF-fed minocycline-treated rats’ intake to that of the control rats. This occurs with restoration of the Firmicutes to Bacteroidetes ratio to a level comparable to that of the chow animals, prevention of the HF-induced decrease in *Bacteroidales* and *Sphingobacteriales*, and significant reduction in *Erysipelotrichales* [53]. Administration of oligofructose, a beneficial prebiotic fermented by intestinal microbes [65], restores populations of *Akkermansia muciniphila* in DIO mice and normalizes BW [66]. In addition to preventing weight gain, probiotics have been found to promote weight loss in mice fed a HF diet for 12 weeks. In these animals, supplementation with a probiotic containing *Lactobacillus rhamnosus*, *Lactobacillus acidophilus*, and *Bifidobacterium bifidum* for 5 weeks decreases BW and food intake [67]. In young men, supplementation with a probiotic along with the initiation of a HF diet (55% kcal from fat, 25% of kcal from saturated fat) reduces the amount of weight gained over 4 weeks [68]. Based on these data, microbiome alterations appear sufficient to alter food intake and necessary for HF-diet-induced increases in intake.

## 3. Microbiome Composition Influences Peripheral Intake Mechanisms

The presence of food in the GI tract leads to the release of satiety signals, such as CCK, by enteroendocrine cells (EECs) that can signal via the vagus nerve to regulate food intake, particularly meal size [23,24]. There is evidence that GI bacterial makeup modulates several aspects of this gut–brain communication.

### 3.1. GI Satiety Peptide Expression/Release

An individual’s gut microbiota may affect regulation of meal size via modulation of GI satiety peptide expression and release. GF mice, when compared to conventional mice of similar body weight, display decreased intestinal expression of CCK peptide [36]. Further, fructose malabsorption induces microbiome alterations, which, in mice, is associated with changes in CCK expression and secretion [69]. Ketohexokinase (KHK)-knockout mice are a model of fructose malabsorption. KHK catalyzes fructose phosphorylation and KHK deletion prevents the upregulation of GLUT5, a fructose transporter [69]. KHK-KO mice do not absorb most fructose, and feeding these animals a diet of 20% fructose leads to increased fructose concentration in the colon and alterations in microbiome composition, including increased relative abundances of Actinobacteria (families *Coriobacteriaceae* and *Corynebacteriaceae*), Bacteroidetes and *Lactobacillaceae* (particularly *Lactobacillus johnsonii*); and decreased Proteobacteria (family *Desulfovibrionaceae*) [69]. These alterations are accompanied by a significant increase in CCK-positive EECs, which can be prevented via antibiotic administration, demonstrating that the microbiota is necessary for fructose malabsorption-induced alterations in CCK release [69]. In addition to modulating CCK expression, microbiome may influence CCK release. In the murine EEC line STC-1, application of certain fatty acid metabolites produced by commensal lactic acid bacteria results in increased CCK release [70].

The microbiota’s influence on gut satiety peptides is not limited to CCK. Glucagon-like peptide (GLP) 1 is an incretin released from intestinal L-cells that decreases food intake via a vagally mediated pathway [71]. There is evidence that short chain fatty acids (SCFAs) produced by a healthy microbiome [72] influence GLP-1 release [15]. Application of SCFAs-acetate, propionate, and butyrate-to-mouse colonic cell cultures leads to increased secretion of GLP-1 through activation of the free fatty acid receptor (FFAR) 2, a nutrient-sensing G-protein coupled receptor [15]. This suggests that bacterial metabolites may be able to directly interact with L-cells to regulate GLP-1 release [15]. SCFAs are produced through fermentation of soluble fibers such as inulin, and while most gut bacteria can produce acetate, there are specific taxa that produce propionate and butyrate [73]. Propionate producers include Bacteroides spp., *Salmonella* spp., *Megasphaera elsdenii*, *Coprococcus catus*, and *Ruminococcus obeum*; and butyrate producers include *Anaerostipes* spp., *Roseburia* spp., and *Coprococcus comes*, *eutactus*, and *catus* (this list is non-exhaustive) [73]. Supplementation of inulin and other prebiotic fibers has been shown to prevent hyperphagia associated with energy-dense diet consumption in rodents [74,75] as well as increased cecal and portal GLP-1 concentrations in rats fed standard chow [76]. Similarly, rats pretreated with 35 days of oligofructose supplementation consumed less food, gained less weight, and had nearly twice the expression of portal and colonic GLP-1 when switched to a HF diet compared to control rats [74]. Oligofructose supplementation for 3 weeks in chow-fed rats leads to a decrease in intake accompanied by increased cecal and portal concentrations of GLP-1 and peptide YY (PYY), another anorexigenic gut peptide [76]. A study in humans found that acute supplementation of inulin-propionate ester increased plasma GLP-1 and PYY and was associated with decreased food intake at a meal post-supplementation when compared to controls [77], showing that propionate has acute effects on food intake. Interestingly, prebiotic supplementation results in increased colon length [75,76] compared to non-supplemented controls, which led the authors to conclude that GLP-1 increase may be due in part to an increased number of secretory cells [76]. However, GF mice with significantly decreased intestinal expression of satiety peptides (CCK, GLP-1, and PYY) exhibit increased cecal and decreased ileal counts of EECs compared to conventional mice [36]. A study found that GF mice have altered ileal expression of genes related to vesicle organization in L cells that was accompanied by increased GLP-1 in ileal L cells, while the colonic transcriptome was not significantly altered [78]. The authors suggest that this is due to the colonic mucus barrier, which prevents bacteria from coming into direct contact with EECs, while microbes in the ileum may come into direct contact with the mucosa [78]. Studies have also found that GF mice have more EECs in the colon compared to conventional mice [36], or mice colonized with *Bacteroides thetaiotaomicron*, which may be due to differences in neurogenesis [79]. Microbiota may influence GLP-1 release through yet another mechanism. Administration of *Akkermansia muciniphila* as a probiotic in obese mice restores levels of acylglycerols in the gut [66]. Acylglycerols are products of fat digestion and components of the endocannabinoid system, and one acylglycerol, 2-oleoylglycerol, stimulates L-cells to secrete gut peptides, including GLP-1, through stimulation of a G-protein-coupled receptor [80] (Table 1).

### 3.2. CCK and Leptin Signaling

In addition to affecting gut peptide expression and release [15,69,81], there is evidence that changes in an individual’s microbiota composition modulate vagal afferent sensitivity to gut-originating satiety signals, particularly CCK [14]. CCK is released from the proximal GI tract (duodenum and early jejunum) in response to long-chain fatty acids [82] and amino acids [83], and acts on VANs to promote meal termination [84]. Colonization of microbiota-depleted rats with a HF-type microbiome is sufficient to reduce CCK-induced satiety in the receiver animals [16]. Conversely, it has been shown that preventing HF diet-driven dysbiosis through prebiotic supplementation prevents HF diet-induced loss in CCK signaling [52], demonstrating that changes in microbiome composition are also necessary for HF diet-induced alterations in CCK signaling.

VAN sensitivity to CCK may be altered through bacterial metabolites and their effect on leptin signaling. Leptin, an anorexigenic adipokine, is released from adipocytes in proportion to fat mass [39] and enhances CCK signaling [85]. Peripheral leptin resistance has been linked to a reduction in CCK sensitivity and increased intake [14,33]. Lipopolysaccharide (LPS), a pro-inflammatory by-product of Gram-negative bacteria, increases in circulation in DIO rodents [14,86]. Cecal and serum concentrations of LPS are increased in animals fed both LFHS and HFHS diets [17]. In rats, chronic low-dose administration of LPS, resulting in serum levels comparable to those seen in HF-fed animals, leads to VAN leptin resistance and decreased sensitivity to CCK [14]. LPS leads to Toll-like receptor (TLR) 4 activation at the level of the nodose ganglion (NG), where VAN cell bodies are located, which subsequently increases SOCS3 protein levels. SOCS3 inhibits activation of the leptin receptor, potentially abolishing the synergistic effect of leptin on CCK sensitivity and decreasing feeding suppression following CCK injection [14].

### 3.3. Inflammation

In rats, DIO is characterized by a leaky gut and low-grade inflammation, potentially driven by bacterial products such as LPS [51]. GI-originating inflammation may play a key role in mediating HF diet-associated alterations in post-ingestive gut–brain signaling. Interestingly, HF feeding rapidly activates microglia-like cells in the NG [17,87], and this may be mediated by microbiota. Microglia are the resident macrophages of the CNS [88], and chronic activation of microglia causes inflammation [89]. Colonization of microbiota-depleted rats with a HF-type microbiome leads to an increase in positive staining in the NG for the pan-microglia and monocyte marker-ionized calcium binding adaptor molecule (Iba) 1 [16], while administration of antibiotics [17] or prebiotics [52] prevents Iba1^+^ cell recruitment along the gut–brain axis. In the CNS, microglia alter synaptic function and axonal growth in response to bacterial products by releasing cytokines [90,91,92], and HF diet-driven microglial activation is associated with inflammation-mediated neuronal death [93]. It is therefore possible that microbiota-driven recruitment of Iba1^+^ cells in the NG has a deleterious effect on VAN survival. Co-culture of VANs with Gram-negative bacteria isolated from HF-fed rats (specifically *Proteus mirablis* of the order *Enterobacteriales* mentioned previously) leads to a dramatic decrease in viable neurons, suggesting that bacterial products can directly influence VAN survival [53]. Further, increased serum LPS and decreased innervation of the cecum was found in rats fed a HF diet [17]. These data would suggest that, in addition to altering vagal afferent signaling, an individual’s microbiota composition could also affect the structural integrity of the gut–brain axis. A decrease in VAN number may explain the reduction in c-fibers observed in the NTS of GF rats conventionalized with a HF-type microbiome [16]. At the level of the NTS, c-fibers are predominantly of vagal origin [94]. Conversely, administration of a broad-spectrum antibiotic [17] or prebiotic [52] concomitant with HF diet introduction can prevent both dysbiosis and c-fiber withdrawal from the NTS.

## 4. Microbiota Influences Central Intake Mechanisms

### 4.1. Neuroinflammation

Besides altering sensitivity to leptin and CCK, bacterial inflammatory products produced by the obese-type microbiome are linked to inflammation and loss of function in key brain regions involved in food intake—the NTS, as previously discussed, and the hypothalamus [95,96,97]. The hypothalamus contains key anorexigenic and orexigenic neuronal populations involved in regulating appetite and energy expenditure. Signals related to energy stores within the body, especially leptin, can modulate neuropeptide expression and release within the hypothalamus to regulate energy homeostasis. Inflammation and cytokine signaling interfere with leptin sensitivity in neurons [98]. Conventionally raised mice exhibit increased hypothalamic SOCS3 expression as well as decreased suppression of orexigenic mRNA (*Npy* and *Agrp*) in response to intraperitoneal leptin injection compared to GF mice [99], hinting that the presence of certain bacterial taxa may interfere with hypothalamic leptin sensitivity. Increased bacterial LPS may play a role. Female rats fitted with slow-release pellets set to deliver a daily low (53 µg/day) or high (207 µg/day) dose of LPS were fed a chow or 60% HF diet for 8 weeks [100]. At the end of the study, both LPS groups had gained more weight and consumed more food than the control vehicle pellet group. LPS groups dose-dependently increased expression of IL-1β in the hypothalamus, while increasing the expression of orexin, a neuropeptide that increases food intake, in the low-dose group [100]. In DIO mice, increased TLR4 and IL-6 mRNA expression in the hypothalamus is associated with decreased leptin-induced STAT3 phosphorylation and a failure to decrease food intake in response to intraperitoneal leptin injection [67]. Supplementation with a probiotic containing *Lactobacillus rhamnosus*, *Lactobacillus acidophilus*, and *Bifidobacterium bifidum* results in decreased BW and food intake in DIO animals, as well as normalization of TLR4 and IL-6 mRNA levels in the hypothalamus, and restoration of leptin-induced pSTAT3 expression [67]. Similar preservation of leptin signaling has been observed with *Lactobacillus rhamnosus* supplementation alone [101], demonstrating that the presence or absence of certain bacterial taxa can modulate hypothalamic leptin signaling.

Oxidative stress is another inflammatory measure that is increased in DIO rats and may be related to microbiome composition. In a study by Fouesnard et al., rats were placed on either a chow or high-energy Western diet (WD; 45% fat) for 6 weeks [95]. WD-fed rats exhibited hyperphagia in the first week of feeding, increased weight gain, and adiposity. Metabolomic changes were observed in the hypothalamus within 2 h of diet introduction, and these changes persisted after the first day of feeding [95]. Specific alterations included hypothalamic redox homeostasis (increased oxidized glutathione, among other measures, suggests increased oxidative stress) and cell membrane remodeling processes. Similarly, cecal microbiome composition was significantly altered within hours of diet introduction, with WD feeding leading to decreased α-diversity and increased Proteobacteria relative abundance, particularly the *Desulfovibrionaceae* and *Tannerellaceae* families, as well as decreased *Lactobacillaceae* relative abundance. Cecal metabolites also correlated with hypothalamic metabolites—one notable association was seen between oxidative stress and indices of α-diversity. This demonstrates an immediate pro-inflammatory microbiome shift within 1 day of WD feeding that coincides with alterations in hypothalamic oxidative stress and hyperphagia [95]. Interestingly, in conventionally raised, but not GF, rats, RNA expression of superoxide dismutase 2, glutaredoxin, and IL-6 are increased after 2 days of WD feeding, demonstrating that the microbiota is necessary for the early pro-inflammatory effects of WD in the hypothalamus.

### 4.2. Reward Pathways

External factors can override homeostatic regulation of intake, including food availability, social and contextual cues, and palatability [71]. The ventral tegmental area (VTA) contains receptors for peripheral energy signals, including ghrelin, insulin, and leptin [102]. It also receives input from the hypothalamus and the NTS [102]. Optogenetic activation of VANs that innervate the upper GI tract stimulates reward-associated behavior such as self-stimulation, place preference, and flavor conditioning, and is sufficient to increase dopamine (DA) levels in the dorsal striatum [23]. Further, ablation of the vagal–parabrachial–nigrostriatal pathway abolishes conditioned preference for gastric infusion of high-calorie nutritive lipids over low-calorie nutritive lipids, while vagal deafferentation alone decreases conditioned preference and avoidance learning in a number of other tests [23]. As microbiota has been found to modulate vagal signaling, changes in bacterial composition are expected to modulate central mechanisms regulating reward.

GF and antibiotic-depleted mice exhibit alterations in dopaminergic reward pathways [19]. GF mice have increased DA turnover in the striatum and lower expression of D1 receptor mRNA in the striatum and NAc [103], a region involved in food-seeking behavior [104], and display increased preference for even low concentrations of intralipid compared to conventional mice [36]. Antibiotic administration has resulted in increased L-3,4-dihydroxyphenylalanine (L-DOPA) in the amygdala of young mice [105] and decreased DA turnover in the amygdala and striatum in rats, suggesting that microbiota modulates DA neurochemistry in rodents. Colonization with fecal contents from mice with chronic ethanol exposure leads to depressive and anxiety-like behaviors similar to those evident in withdrawal [20], and administration of SCFAs to mice previously exposed to a small dose of cocaine abolishes conditioned place preference [106], which suggests that microbiota is directly involved in modulating addiction-like behaviors and may be relevant in food addiction. These data suggest that individual microbiota may affect food intake not only through homeostatic mechanisms, but also through regulation of the reward pathway and hedonic perception. There are studies emerging that support this hypothesis.

In adolescent rats with intermittent daily access to HFHS diet, overall energy intake increases and monoamine gene expression is altered in the hippocampus and prefrontal cortex, and these alterations correlate with bacterial relative abundances [56]. Specifically, prefrontal cortex expression of monoamine oxidase A is positively associated with an unspecified genus of the *Lachnospiraceae* family, while expression in the hippocampus is associated with a number of other families, including unspecified *Bifidobacteriales*, *Bifidobacteriaceae*, unspecified *Bacteroidales*, *Rikenellaceae*, *Lachnospiraceae*, *Ruminococcaceae*, and *Erysipelotrichaceae* [56]. Microbiota may play a role in food preferences as well. Increased preference for sucrose is evident in mice undergoing social stress, and this increased preference is abolished by SCFA supplementation, suggesting that microbiota modulates stress-induced sucrose preference via SCFA production [107]. In rats, chronic consumption of HFHS diets leads to decreased motivation to lever press to receive a sucrose pellet [108]. Fructo-oligosaccharide introduced along with the initiation of HFHS feeding restores motivation for the sucrose pellet; however, supplementation beginning after 10 weeks of HFHS diet exposure is not able to rescue this measure of motivational behavior [108]. Further, supplementation leads to decreased preference for HFHS foods compared to rats without supplementation [108].

Low- and no-calorie artificial sweeteners are another point of contention in terms of their impact on reward and intake, as it has been found that some sweeteners, such as stevia, are metabolized by gut microbiota [109]. While short- and long-term studies in humans do not show that artificial sweetener intake leads to compensatory overeating [110], there is evidence in both humans and rodents that sweetener intake causes alterations in reward pathways. In humans, ingestion of sucralose has significantly different effects in VTA activation compared to glucose or sucrose [111]. Rats exposed to a chronic low dose of rebaudioside A (RebA), a stevia glycoside, exhibit decreased tyrosine hydroxylase and dopamine transporter (DAT) mRNA expression in the NAc [112], which can be rescued by prebiotic oligofructose supplementation [112]. These data suggest that artificial sweeteners that are metabolized by microbiota may alter reward signaling. It should be noted that the evidence is limited as research in this area is still emerging (Table 2).

## 5. Summary

Microbiota exerts an undeniable influence in the regulation of food intake through central and peripheral mechanisms, including satiety peptide release and signaling, inflammation, and modulation of reward pathways. Microbiota-driven changes in gut–brain signaling may be linked to alterations in both homeostatic and hedonic regulation of feeding. Preventing adverse microbial alterations via supplementation of prebiotic fibers and probiotics can successfully prevent hyperphagia in animal models, especially when introduced concomitantly with a dietary challenge. However, changes to the gut–brain axis may have long-term consequences on feeding behavior. HF or HS feeding [17] and HF-type microbiome alone [16] lead to withdrawal of vagal c-fibers from the NTS. This remodeling coincides with onset of weight gain and hyperphagia [38]. Nerve injury-induced [113] or diet-induced [53] vagal withdrawal can be followed by NTS reinnervation (sprouting). Crucially, reinnervation does not appear to restore function in HF-fed rats, as animals remain hyperphagic [38,53], suggesting that gut–brain function may be permanently affected in obesity. It is still unclear if microbiome-based therapy could restore gut–brain signaling in obesity. While weight loss in both humans and rodents is associated with microbiome composition alterations [114,115], there is limited evidence that restoring microbiome in obese individuals can lead to weight loss. Probiotic use may be circumstantially associated with weight loss [116], but no causal link has been established. Most animal studies in the realm of obesity research focus on preventing weight gain when introducing a HF diet and initiate treatments such as pre- and probiotics concomitantly [117,118,119,120]. While helpful when flushing out the etiology of obesity, these studies do not determine whether modulation of an individual’s microbiota can successfully and effectively decrease food intake and promote clinically meaningful weight loss. More studies should be executed in which pre- or probiotics are supplemented to DIO animals to determine whether microbiome composition can be restored to a pre-obese state, or if modulation is associated with weight loss. Such studies would help determine whether the microbiota is an appropriate target to promote healthy eating behavior and weight loss.

## Figures and Tables

**Table 1:** Gastrointestinal (GI) satiety peptides and their association with microbiota.

Satiety Peptide	Association with Microbiota
CCK	VANs exhibit decreased CCK sensitivity when the GI tract is colonized with HF-type microbiome [16].
GLP-1	SCFAs produced when microbiota ferments soluble fibers may promote GLP-1 secretion [15,76].
PYY	Rats fed soluble fiber also exhibit increased PYY levels in the GI tract [76].

**Table 2:** Bacteria associated with food intake alterations. It is important to note that there is currently insufficient evidence to consistently link specific bacterial species to altered intake. Further, these findings have only been demonstrated in rodent models and are not applicable in humans.

Bacteria	Intake Alterations
*Lactobacillus rhamnosus*, *Lactobacillus acidophilus*, and *Bifidobacteria bifidum*	5 weeks of supplementation decreased hypothalamic inflammation, food intake, and BW compared to DIO animals without the supplement [67]
*Akkermansia muciniphila*	May promote gut peptide release through increasing acylglycerols in the gut [66]
Gram-negative bacteria	Produce LPS, which can ○ reduce VAN sensitivity to leptin and CCK [14] ○ hypothalamic inflammation and BW [100] May decrease VAN survival (specifically, Proteus mirablis) [53]
